# Immunologically Active Components in Human Milk and Development of Atopic Disease, With Emphasis on Food Allergy, in the Pediatric Population

**DOI:** 10.3389/fped.2018.00218

**Published:** 2018-08-07

**Authors:** Puja S. Rajani, Antti E. Seppo, Kirsi M. Järvinen

**Affiliations:** Division of Pediatric Allergy and Immunology and Center for Food Allergy, University of Rochester School of Medicine and Dentistry, Rochester, NY, United States

**Keywords:** breast milk composition, breast feeding, atopic development, IgA, breast milk microbiome, cytokines, human milk oligosaccharides (HMOs), fatty acids

## Abstract

Breast-feeding is currently recommended to prevent the development of allergic diseases; however, data are conflicting and mechanisms are unclear. The immunomodulatory composition of human milk is poorly characterized and varies between mothers. We and others have shown that high levels of human milk IgA and certain cytokines and human milk oligosaccharides are associated with protection against food allergy in the infant, but it is unclear whether they are responsible for or simply biomarkers of the vertical transfer of protection. Because human milk has pre- and probiotic properties, the anti-allergy protection afforded by human milk may be due to its control on the developing gut microbiome. In mice, murine milk IgA supports gut homeostasis and shapes the microbiota, which in turn diversifies the intestinal IgA repertoire that reciprocally promotes the diversity of gut microbiome; these mechanisms are poorly understood in humans. In addition, several human milk bioactives are immunostimulatory, which may in part provide protection against allergic diseases. The regulation of immunologically active components in human milk is incompletely understood, although accumulating evidence suggests that IgA and cytokines in human milk reflect maternal exposures. This review summarizes the current literature on human milk components that have been associated with protection against food allergy and related allergic disorders in early childhood and discusses the work relating to regulation of these levels in human milk and possible mechanisms of action.

## Introduction

**Figure 1:**
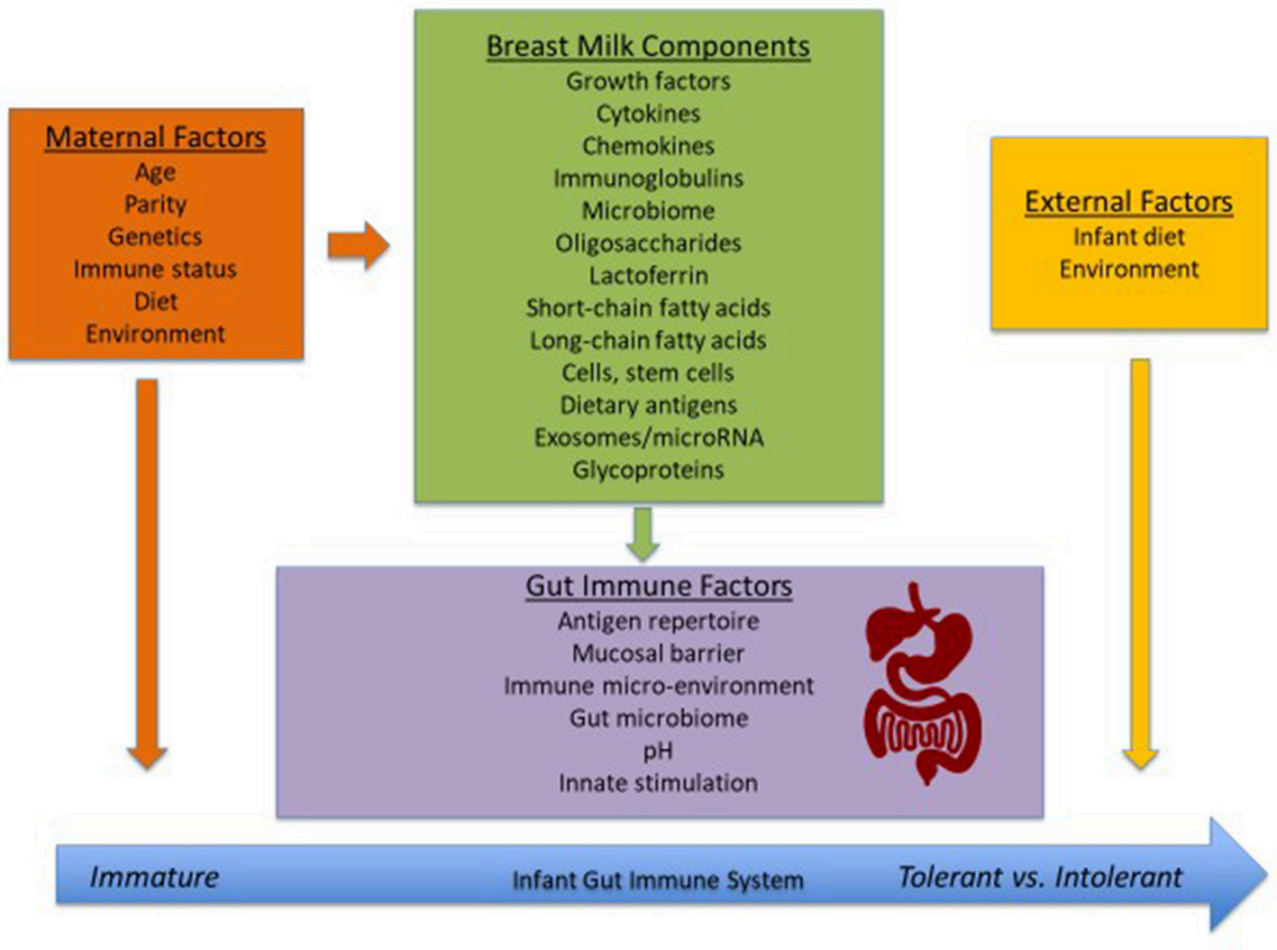
Factors that influence the development of the neonatal immune system.

Breast-feeding is a natural process of providing nourishment to offspring. Human milk is the optimal source of nutrition for term infants during the first 6 months of life as it provides nutrients, antimicrobial factors, and exposure to important immunomodulatory factors infants need to grow, develop, and thrive (1). There are various studies showing that human milk provides defense against infections and development of allergic disease (2, 3). The first few months of life are a crucial window in which the still-developing infant immune system can be influenced, with breast-feeding allowing for continued exposure to the mother's immune system. This can impact oral tolerance induction and development of allergy (Figure 1). However, the immunomodulatory composition of human milk is poorly characterized and varies between mothers.

Many studies have been published investigating the effect of breastfeeding on atopic diseases, though conclusions from these studies were conflicting, with some authors claiming a protective effect, some remaining undecided, and a few even suspecting that breastfeeding might promote the development of atopic diseases (4–6). Systematic reviews and meta-analyses have concluded an overall protective effect of breastfeeding against atopic dermatitis, wheezing/asthma, allergic rhinitis and cow's milk allergy (CMA) in early childhood (7–10), and breast-feeding is currently recommended to prevent allergic diseases (11). A multidisciplinary review of the literature from 1966-2001 by van Odijk et al. reviewed 132 articles discussing early feeding methods and outcome of atopic disease (7). Only 56 of these articles were conclusive and the conclusion of the reviewers was that breastfeeding is protective of atopic diseases (asthma, recurrent wheezing, atopic dermatitis), and the protective impact is stronger in children with atopic heredity. The review also concluded that exposure to small doses of cow's milk during first days of life predisposes to cow's milk allergy (CMA), and in children with atopic heredity, breastfeeding and extensively hydrolyzed formula protect against CMA. A meta-analysis by Gdalevich et al. in 2001 showed that at least 3 months of exclusive breastfeeding protected from eczema and asthma in children with a family history of atopy (12, 13). Development of food allergy was not assessed. This has been reproduced in various other observational studies from Australia, Sweden, and Denmark. (14–16). The Promotion of Breastfeeding Intervention Trial (PROBIT), a large randomized trial from Belarus, was able to promote breastfeeding duration and exclusivity of breastfeeding at 16 hospitals and found that at these sites, infants had fewer gastrointestinal infections and lower incidence of eczema in the first year of life (17). However, the follow-up at 6 years of this same cohort showed a lack of protective effect with this intervention on asthma, eczema, or hay fever (5). The American Academy of Pediatrics Committee on Nutrition and Section on Allergy and Immunology published a clinical report in 2008 concluding that there is evidence that breastfeeding until 4 months, compared with feeding formula made with intact cow's milk protein, prevents (or delays) the occurrence of atopic dermatitis, wheezing and cow's milk allergy in early childhood (9). Interestingly, Katz et al. reported in 2010 in a large-scale prospective population-based study that early exposure to cow's milk protein as supplementation to breastfeeding might prevent IgE-mediated cow's milk protein allergy (18). The Cochrane Database Systematic Review in 2012 by Kramer and Kakuma (3) concluded that with breastfeeding *beyond* 3-4 months, there is no significant reduction in risk of atopic eczema, asthma, or other atopic outcomes demonstrated in studies from Finland, Australia, and Belarus. This was confirmed to be the case for eczema in the retrospective ISAAC Phase Two Study of >51,000 children randomly selected in 21 countries (19). The most recent systematic review by Lodge et al. from 2015 showed the protective effect of more vs. less breastfeeding against risk of asthma in children 5–18 years, especially in lower income countries, and against allergic rhinitis in children ≤ 5 years (10). There was a significant effect of protection against eczema for children ≤ 2 years by exclusive breastfeeding for 3-4 months. Estimate for an effect of breastfeeding on food allergy had high heterogeneity and low quality. Most recently, a retrospective study in 2016 from Japan noted that cow's milk formula exposure during the first 3 months of life may also have a protective effect on CMA (20). However, data are conflicting, especially given the lack of randomized controlled trials and varied definitions of breastfeeding and allergic outcomes. Unfortunately, most studies have been underpowered for food allergies or not assessed at all due to methodologic problems of making the firm diagnosis. However, among all the atopic diseases, breastfeeding may have the most impact on development of oral tolerance to foods, which develops in the gastrointestinal tract. Epidemiologic studies have not accounted for the human milk composition, which varies from one mother to another, and may be a remarkable confounder impacting its protective properties.

Human milk impacts the development of the infant gut microbiome, along with other maternal and environmental factors. At birth the infant transitions from a highly regulated maternal, microbiota-scarce environment to becoming colonized with *ex utero* microbiota (21). With vaginal birth, the infant microbiota originates mainly from the mother's intestine, vagina and skin, while the hospital environment and the mother's skin provide the first colonizing microbes with C-section birth (21–23). The bacterial colonization of the newborn intestine may contribute to development of the neonatal immune functions or susceptibility to immune-mediated disorders in early (and later) life (6, 24, 25). Evidence from both animal (26) and human studies (27–31) have reported that gut dysbiosis precedes the development of atopy, atopic eczema and food allergy/sensitization. In the past year, several studies have linked the importance of gut microbiome and food allergy. Kourosh et al. sought to better understand fecal microbiome in children with IgE mediated food allergy and were able to show that there were significant differences in microbial composition amongst food-allergic children, especially in the *Clostridia* class, compared with healthy siblings and healthy children (32). Fieten et al. looked for differences in fecal microbiome in children with or without food allergy in the setting of atopic dermatitis (33). Their pilot study showed significant differences in the microbiome profile between these two groups, specifically with *Bifidobacterium breve, Bifidobacterium pseudocatenulatum, Bifidobacterium adolescentis, Escherichia coli, Faecalibacterium prausnitzii*, and *Akkermansia muciniphila*. Finally, Fazlollahi et al. looked at the role of gut microbiota in egg allergic children and found a distinction in diversity of microbial flora compared to non-food allergic controls (34). While this data is important for our discovery of the end outcome of atopy, the specific human milk components on microbiome and atopy development are discussed in this review.

Human milk originates in the lactating mammary tissue. Milk lipid, lactose, and the majority of milk proteins are produced in the lactating cells (35). Human milk contains immune cells, immunoglobulins, cytokines, chemokines, growth factors, lactoferrin, oligosaccharides, enzymes (peroxidases, lysozymes), secretory components, and hormones, along with foreign food antigens, bacteria and viruses (6, 36). Several of these bioactive factors have been assessed in relation to development of allergies in the infant, and many of these immunologically active factors in human milk are missing in processed cow's milk and infant formulas, in which the whey to casein ratio is markedly lower than in human milk (37, 38). This review summarizes the current literature on human milk components that have been associated with protection against food allergy and related allergic disorders in early childhood and discusses the work relating to regulation of these levels in human milk and possible mechanisms of action.

## Cytokines, chemokines, and growth factors

Cytokines, which include chemokines, interleukins, interferons, and growth factors, are signaling molecules that function in cellular communication. Human milk is a rich source of immunostimulatory and immunoregulatory cytokines (6, 39). There is variation in the concentration of cytokines among mothers, and overall concentrations for several of those are relatively low in human milk, causing debate in the clinical significance of cytokine levels on health outcomes. Some of the variation in cytokine levels is thought to be due to varying maternal (microbial) exposures. Milk interleukin (IL)-10, interferon (IFN)-γ (40) and transforming growth factor (TGF)β (41) levels have been shown to vary depending on mothers' country of residence, and country of birth (42), and TGFβ as an example is in human milk at a biologically meaningful concentration.

TGFβ is an important regulatory cytokine involved in suppression of both Th1 and Th2 pathways, and is the molecule that has been most studied. The three isoforms of TGFβ combined make it the most prevalent cytokine in human milk, with the most abundant being TGFβ-2 (43, 44). Immunomodulatory cytokines in murine milk, including TGFβ have been shown to influence the development and maturation of the mucosal immune system in neonatal mice and to be associated with the protection against allergic asthma (45). Some studies have confirmed that milk TGFβ is immunologically active, and involved in the induction of oral tolerance, perhaps by inducing increased production of specific IgA (46, 47). Alternatively, TGFβ-2 has been shown to induce maturation of immature intestinal epithelial cells (48). Protection induced by human milk TGFβ has especially been noted in the development of atopic dermatitis (43). This was supported in a review in 2010 by Oddy and Rosales of twelve human studies that determined that 67% of the studies showed a positive association of TGFβ-1 or TGFβ-2 preventing atopic outcomes in infancy and early childhood (49). The study concluded that TGFβ is likely essential in the development of immune responses in infants and may provide protection against adverse immunological outcomes (49). Overall, however, there is conflicting data regarding the role TGFβ in the development of atopic disease in humans (41, 50–56). Most recently, a study by Morita et al. showed that lower concentration of TGFβ-1 in human milk at 1 month, but not TGFβ-2, may be linked to development of eczema (57). In another study of food allergy, the concentration of TGFβ-1 in colostrum from mothers of infants with IgE-mediated cow's milk allergy was lower than from mothers of infants with non-IgE-mediated cow's milk allergy; however, the levels in healthy controls were found in between (58). The studies are summarized in Table 1. A recent study showed that human milk TGFβ was associated with increased richness, evenness and diversity of infant gut microbiome composition (61).

**Table 1:** Studies pertaining to TGFβ in human milk and development of atopic disease.

**Study**	**Year**	**Location**	**Size**	**Duration/Age**	**Outcomes**
Kalliomaki et al. (43)	1999	Finland	*n* = 47	Up to 12 months	Increased TGFβ-1 and 2 levels in colostrum were associated with higher post weaning-onset atopic disease
Saarinen et al. (58)	1999	Finland	*n* = 6209	Up to 12.7 months	Increased TGFβ-1 levels in colostrum are associated with infants who develop IgE-mediated cow's milk allergy versus non-IgE-mediated cow's milk allergy; healthy controls were found in between
Bottcher et al. (50)	2003	Sweden	*n* = 53	Up to 2 years	TGFβ-1 and 2 levels were not significantly associated with eczema, salivary IgA, or allergic sensitization
Oddy et al. (59)	2003	Australia	*n* = 243	Infancy	Increased TGFβ-1 is associated with lower risk of wheeze in infancy
Savilahti et al. (51)	2005	Finland	*n* = 4674	Up to age 4 years	TGFβ-1 and 2 levels were not significantly associated with atopy development
Snijders et al. (52)	2006	Netherlands	*n* = 315	Eczema (up to 12 months), Wheezing (up to 2 years), Allergic sensitization (up to 2 years)	No significant association of with TGFβ-1 and development of eczema, wheezing or allergic sensitization
Bottcher et al. (60)	2008	Sweden	*n* = 54 (*L. reuteri*) *n* = 55 (control)	Up to 2 yeas	Decreased TGFβ-2 in colostrum is associated with lower incidence of allergic sensitization and a trend of protective effect on eczema development
Kuitunen et al. (53)	2012	Finland	*n* = 364 (colostrum) *n* = 321 (BM)	At 2 years of age	Increased TGFβ-2 is associated with higher risk of allergic disease and eczema
Ismail et al. (54)	2013	Australia	*n* = 79	Up to 12 months	TGFβ-1 level was not significantly associated with eczema or allergic sensitization
Orivuori et al. (41)	2014	Finland, France, Germany and Switzerland	*n* = 610	Eczema (up to 4 years), asthma (up to 6 years), allergic sensitization (up to 6 years)	TGFβ-1 level was not significantly associated with eczema, asthma, or allergic sensitization
Jepsen et al. (55)	2016	Denmark	*n* = 223	Up to 3 years	TGFβ-1 level was not significantly associated with recurrent eczema or wheeze
Munblit et al. (56)	2017	United Kingdom, Russia and Italy	*n* = 398	Up to 6 months	Increased TGFβ-2 is associated with higher risk of eczema
Morita et al. (57)	2018	Japan	*n* = 43 (eczema) *n* = 53 (control)	Up to 6 months	Lower TGFβ-1 ratio (1-month milk/colostrum) is associated with higher risk of eczema

Emerging data regarding the role of other human milk cytokines and chemokines on allergic disease development has been variable. A summary of association between cytokines and the development of food allergy can be found in Table 2. Earlier studies using ELISA found that levels of IL-4 are lower and IL-8 and CCL5 (RANTES) are higher in human milk from atopic compared to non-atopic mothers (63, 64), though others found that cytokine levels were largely not related to maternal atopy (6, 65). Various studies report low to undetectable levels of other cytokines and chemokines including IFNγ, IL-2, IL-4, IL-5, IL-10, IL-12, IL-13, CCL5, CXCL8, CXCL10, and TNF-α and have found no association with development of atopic disease despite their involvement in immune and intestinal development (39, 50, 53, 55, 66, 67). Pro-inflammatory cytokines, including IL-1β, IL-6, and IL-8 are also found in human milk in low concentrations. IL-6, IL-8, CXCL8, and CXCL10 in human milk have been shown to be affected by factors such as gestational smoking, maternal race, and season (68) and exercise has been associated with elevated levels of IL-1β and IL-17 (69). There are studies showing that some of these cytokine levels in milk may impact allergic outcomes in offspring. Increased levels of IL-1β in human milk have been shown to be associated with protection against eczema (55). Soto-Ramirez et al. showed that IL-5 and IL-13 levels in milk, although extremely low, are risk factors for asthma at 12 months of age (67). CCL5 in milk was the strongest risk factor for development of atopic dermatitis in the study by Ochiai et al. (65). Because food allergy represents a failure in development of mucosal tolerance to foods, immune factors in human milk may have a more direct effect on development of food allergy (62). In fact, our study showed that a panel of pro-inflammatory and regulatory cytokines including IL-1β, IL-6, IL-10, and TGFβ-1 in human milk were associated with protection against CMA (6, 62). These cytokines promote IgA production, Th17 differentiation and microbiota-driven crosstalk between gut macrophages and RORγt^+^ ILC-3 population (70). It is unclear whether these bioactive factors are directly related to protection or whether they are biomarkers of another protective mechanism (6).

**Table 2:** Summary of association between cytokines and the development of food sensitization/allergy.

**Study**	**Year**	**Location**	**Size**	**Duration/Age**	**Cytokines assessed**	**Food allergy development**
Bottcher et al. (50)	2003	Sweden	*n* = 53	Up to 2 years	IL-4, IL-5, IL-6, IL-8, IL-10, IL-13, IL-16, IFN-γ, TGFβ-1, TGFβ-2, RANTES, eotaxin	No significant association
Snijders et al. (52)	2006	Netherlands	*n* = 315	Up to 2 years	IL-12 or TGFβ-1 (IL-10 undetectable)	No significant association
Kuitunen et al. (53)	2012	Finland	*n* = 364 (colostrum) *n* = 321 (3 month HM)	At 2 years of age	IL-10, TGFβ-1	No significant association
Järvinen et al. (62)	2015	Finland	*n* = 145	Up to 2 years	IL-1α, IL-1β, IL-6, IL-10 PDGF-BB, CCL27, VEGF, TSLP, CCL11, CXCL10, and CXCL11, CCL22, TGFβ-1, (TNF-a and -b, CCL1, CCL17, IL-31, eotaxin 3, CXCL9, IL-5, GM-CSF, and IL-12p70 undetectable)	IL-1β, IL-6, IL-10, and TGFβ-1 in human milk showed association with cow's milk tolerance
Munblit et al. (56)	2017	United Kingdom, Russia and Italy	*n* = 398	Up to 6 months	IL-2, IL-4, IL-5, IL-10, IFNγ, IL-12, IL-13, HGF, TGFβ-1, TGFβ-2, TGFβ-3	IL-13 associated with protection, otherwise no significant association

*HM, Human milk*.

Other growth factors have also been shown to be present in high concentrations in human milk, including vascular endothelial growth factor, hepatic growth factor, and epidermal growth factor, though the clinical importance is unknown (56, 62, 71). Most recently, a study was conducted by Munblit et al. in which 398 pregnant/lactating women in the United Kingdom, Russia, and Italy were followed prospectively to look for an association between levels of immune mediators in colostrum/mature human milk and allergic outcomes in infants during the first year of life (56). Hepatocyte growth factor (HGF) in mature human milk was protective against common cold incidence at 12 months. Other study outcomes in infants included eczema symptoms, parental-reported food allergy, and recurrent cough/wheeze at 6 and 12 months of age. Results showed higher levels of IL-13 in the colostrum and mature human milk were protective against parent reported food allergy and eczema respectively. IL-2, IL-4, IL-5, IL-10, IL-12, and IFNγ showed no significant association with eczema, wheeze or food allergy (56).

## Soluble CD14/TLR

Human milk may also influence neonatal microbial recognition by modulating Toll-like receptor (TLR)-mediated responses specifically and differentially (72). Necrotizing enterocolitis has been shown to be reduced in infants who are breastfed, mediated likely via the lipopolysaccharide (LPS) receptor TLR4 preventing mucosal injury and promotion of repair (73). CD14 is the soluble component (sCD14) of the TLR4, which has a role in innate immunity. It binds to LPS from gram-negative bacteria and intestinal enterocytes. The absence of sCD14 reduces the TLR4 response to LPS. Colostrum is rich in sCD14 with levels decreasing over time whereas neonates lack CD14. Soluble CD14 levels have been found to be lower in colostrum and human milk of mothers with children who develop atopy or eczema, sensitization (6, 74). Later studies, however, deny an association between levels of sCD14 and development of atopy (52, 54). In 2015, Savilahti et al. showed that elevated sCD14 in human milk 3 months post-partum was associated with development of IgE-mediated allergic disease by 5 years of age in children who had hereditary risk of atopy, suggesting that sCD14 in milk influences the emergence of allergy in children with atopic heredity (75). This study contrasted with a study by the same group from 2005 that showed sCD14 levels were lower in *colostrum* of mothers with infants developing atopic symptoms and IgE sensitization than of those of infants with no atopy (51). Studies regarding sCD14 in human milk are summarized in Table 3. The conclusions are mixed and there does not appear to be a clear relationship between sCD14 levels in human milk and development of atopic disease.

**Table 3:** Studies pertaining to sCD14 in human milk and development of atopic disease.

**Study**	**Year**	**Location**	**Size**	**Duration/Age**	**Outcomes**
Jones et al. (74)	2002	United Kingdom	Varies (multiple cohorts)	At 6 months	Decreased sCD14 in 3 month HM is associated with higher eczema incidence
Oddy et al. (59)	2003	Australia	*n* = 243	Up to 12 months	sCD14 levels in 2 week HM showed no significant association with infant wheeze
Savilahti et al. (51)	2005	Finland	*n* = 4674	Up to 4 years	Decreased sCD14 levels in colostrum were associated with a higher incidence of allergic sensitization and eczema
Snijders et al. (52)	2006	Netherlands	*n* = 315	Eczema (up to 12 months), wheeze (up to 2 years), or allergic sensitization (up to 2 years)	sCD14 level in 1 month HM was not significantly associated with eczema, wheeze, or allergic sensitization
Ismail et al. (54)	2013	Australia	*n* = 79	Up to 12 months	sCD14 level in 1 and 4 week HM was not significantly associated with eczema or allergic sensitization
Savilahti et al. (75)	2015	Finland	*n* = 260	Up to 5 years	Increased sCD14 level in 3 month HM is associated with higher incidence of allergic sensitization and eczema

*HM, Human milk*.

## Immunoglobulin A (IgA)

The predominant immunoglobulin in human milk is IgA, most of which is in the form of secretory IgA (SIgA), with smaller amounts of IgG and IgM (6, 76). An older study utilized human milk from a prospective birth cohort of 145 mother-infant dyads oversampled for high risk of food allergies and followed for 12-18 months for development of CMA. The study showed that high levels of human milk total (77) and cow's milk-specific IgA (78) were associated with protection against CMA, consistent with other reports (79, 80). While the exact function of IgA in human milk is unknown, it is thought to supplement infant IgA production, which only commences after birth (78, 81). Data from several studies support a role for maternal environment (geographic location, microbial pressure, exposure to farm animals and cats) in driving milk IgA levels and specificity (40, 41, 82). Some studies have shown a link between high IgA levels and protection for the development of atopic dermatitis (41, 51) while other studies show no link between sIgA and development of other atopic diseases (41, 50, 54).

Mucosal IgAs are produced by plasma cells in the gut lamina propria and are transported across epithelial cells by the polymeric immunoglobulin receptor (pIgR) (83). Human milk IgA is produced by mammary gland B cells that have migrated from the mother's intestine via the “enteromammary link” (84, 85), as shown in animal studies (86–89). This is controlled by the mucosal vascular addressin MadCAM-1 or mucosa-associated epithelial chemokine CCL28, which interacts with the gut homing receptor α_4_β_7_ integrin (90) and mucosa-associated CCR10 (91). Consistent with this, in a rabbit model either oral or inhaled RSV resulted in RSV-IgA production in milk, bronchial and enteral secretions, whereas systemic immunization did not (92). Studies in humans (93) showed that oral immunization in women resulted in an increase in plasma cells in milk, but not in saliva or serum, (85). This forms the hypothesis that human milk IgA reflects the antigenic exposure of the mother's gut to dietary proteins as well. Using the cohort mentioned above, it was shown that a strict maternal diet restricting cow's milk was associated with lower levels of sIgA levels in human milk than cow's milk-containing diet (78). This implies that the antigenic stimulation encountered by the maternal gut directs the antibody specificity of human milk (85). In order to further understand the regulation of IgA in milk, epitope-specific binding of IgA in milk was compared to paired maternal serum samples (85). This revealed that IgA in human milk had partially different epitope specificity to cow's milk antigens than IgA in serum, suggesting different pools of antibody-producing lymphocytes controlling serum and human milk antibodies, respectively, and therefore supporting evidence for enteromammary milk. In summary, IgA levels expressed in human milk are influenced by many maternal factors, including diet, location, exposures, microbiota, and likely plays a protective role against development of cow's milk allergy.

## Microbiome

Infant microbiome composition is influenced by breastfeeding (94, 95). Human milk can modify the infant microbiome directly through seeding from the maternal microbiome and through the other effects of human milk. Diversity of the infant gut microbiome develops in the first 2 years of life and *Bifidobacteria* dominate throughout the first year (96). Recent studies have shown that host genetics, prenatal environment and delivery mode can shape the newborn microbiome at birth [reviewed in (97)]. Following this, postnatal factors, such as antibiotic treatment, diet and environmental exposure, further modulate the development of the infant's microbiome and immune system. Living on farms, avoiding antibiotics, vaginal delivery, and other environmental factors leading to greater diversity in the microbiome have been associated with a major reduction in the risk of atopic diseases(98, 99). Several large studies have confirmed the role of breastfeeding in determining the gut microbiome. Initially there is lower microbiome diversity with breastfeeding, as human milk selects for a highly adapted intestinal microbiota, and when breastfeeding is ceased and complementary feeds start, *Lactobacilli, Bifidobacteria*, and *Enterobacteriaceae* are replaced with a microbiota dominated by *Clostridium* and *Bacteroides* species(100–103). The WHEALS birth cohort confirmed that together with the mode of delivery, breastfeeding is one of the most important factors impacting infant microbiome (95). Interestingly, however, only 12–14% of variability was explained by maternal mode of delivery, exposure to pets, demographics and breastfeeding. This may be partly due to the fact that the human milk biologically active components such as IgA and HMOs, which can modulate microbial composition and function, were not specifically considered. Their concentrations vary between mothers, and this variation is not captured in a coarse definition of breastfeeding.

Several culture-dependent and–independent studies have revealed that colostrum and human milk contain a variety of bacterial communities that colonize the infant's gut. The initial studies demonstrated predominance of staphylococci, lactobacilli, streptococci and propionibacterium, and closely related gram-positive bacteria (104). Culture-independent molecular techniques, especially those utilizing 16S rRNA sequencing have confirmed a similar diversity of bacteria, but also presence of several others including Gram-negative bacteria (6, 105–109). Milk bacterial communities vary between mothers but are relatively stable within individuals (106). Human milk microbiota has been shown to act as a source of bacterial species that colonize the infant gut (110), to be but different from skin suggesting an endogenous route for human milk colonization (105, 111). The amount of bacteria ingested by an infant per 800 mL of milk consumed daily is estimated at 1 × 10^5^-1 × 10^7^, though this is likely an underestimation (112). Recently, it was shown that human milk provides a source of about one-fourth of infant gut microbiota (113).

## Human milk oligosaccharides

Human milk oligosaccharides (HMOs) provide the main substrate for an infant's gut microbiota during exclusive breastfeeding, particularly promoting bifidobacteria and Bacteroides (114–116). Some HMOs have anti-inflammatory properties, and support maturation of the gut mucosal immune system (117). Some also have an inhibitory effect on intestinal cell growth (118), and some bind to dendritic cells through the lectin receptor DC-SIGN (119) inhibiting HIV transfer to T-cells. These oligosaccharides are not digestible by the infant and are extensions of lactose generated by the action of a series of glycosyltransferases. For fucose, two fucosyltransferases FUT2 (secretor gene) and FUT3 (Lewis gene) are implicated. Depending on the Lewis blood group and secretor status, different enzymes are available for the synthesis of HMOs. As a result, human milk from different mothers have significant variations in qualitative and quantitative composition of HMOs. HMO composition is relatively stable during the course of lactation, although it is not known whether minor daily variations are due to the mother's diet (120). This heterogeneity implies that some breast-fed infants are not being exposed to certain structures. Non-secretor mothers, lacking a functional FUT2 enzyme (FUT2−/−), represent 15-25% of mothers depending on their ethnic background (121, 122), and their milk is missing all alpha-2 linked fucose oligosaccharides (21). Infants fed by non-secretor mothers are delayed in establishment of bifidobacteria-laden microbiota (123). Differences in HMOs have also been associated with susceptibility to infectious gastroenteritis (124, 125) and HIV (126–128). In our previous studies, certain HMO profiles were associated with protection against cow's milk allergy (129). Infants who received human milk with low Lacto-N-fucopentaose (LNFP) III concentrations were more likely to become affected with CMA when compared to those receiving milk with high levels (*p* = 0.00036, odds ratio 6.7, 95% CI 2.0–22). Two other studies have assessed the association between HMO and atopic diseases. A study that followed 20 infants for the first 18 months for development of FA, and measured HMOs using HPLC was powered to only find major effects, and indeed did not find a significant difference in HMOs between mothers of allergic and non-allergic children (130). In a second study, infants fed by non-secretor mothers had delayed development of bifidobacteria-laden microbiota (123) and if also born via c-section had a higher risk to manifest IgE-associated eczema (21). However, development of food allergy or composition of individual HMOs were not assessed. These data support the role of HMOs in protection against CMA, possibly through their effect on infant gut microbiome. Most recently, the Canadian Healthy Infant Longitudinal Development (CHILD) study, compared HMO profiles with food sensitization at 1 year of age (131). The study found that lower risk for food sensitization was associated with higher concentrations of fucosyl-disialyllacto-N-hexaose (FDSLNH), lacto-N-fucopentaose II (LNFPII), lacto-N-neotetraose (LNnT), lacto-N-fucopentaose I (LNFPI), sialyllacto-N-tetraose c (LSTc), and fucosyllacto-N-hexaose (FLNH), and relatively lower concentrations of lacto-N-hexaose (LNH), lacto-N-tetraose (LNT), 2′-fucosyllactose (2′FL), and disialyllacto-N-hexaose (DSLNH). Further investigation into HMO composition is necessary to better understand the role of HMOs in pathophysiology and possibly future therapeutics for prevention of atopic disease.

## Fatty acids

Milk lipids are principal macronutrients in human milk and studies have shown that milk from atopic mothers varies in fatty acid content. Polyunsaturated fatty acids (PUFAs), more specifically the omega-3 (ω-3) fatty acids, e.g., docosahexaenoic (DHA) and eicosapentaenoic (EPA), have been recently shown to have anti-inflammatory effects in chronic inflammatory diseases, such as asthma (132). On a maternal fish oil supplementation trial, omega-3 PUFA levels were positively associated with IgA and sCD14 levels, suggesting a relationship between fatty acid status and mucosal immune function (133). Another study has shown that atopic mothers' milk has lower levels of n-3 long-chain PUFA at 1 month of lactation than non-atopic mothers (134). Overall, the studies examining the fatty acid profile in human milk as a risk factor for subsequent atopic disease have been mixed, though generally found that n-3 PUFAs in human milk possibly protect against atopic diseases (134–139). The conflicting findings may be due to the complex interactions between different fatty acids types and the divergent functions on immune system based on the dose (6, 140).

More recently, the short-chain fatty acids (SCFAs), including acetate, butyrate and propionate, have been demonstrated as possibly important mediators of allergic inflammation. Inflammation is likely a by-product of the metabolic activity of gut microbiota given that SCFAs are altered in children who are or become overweight or atopic (141). SCFAs are the first metabolites produced by the gut microbiota of newborns, with synthesis increasing rapidly after birth (142). As commensal microbiome has been shown to be protective against food sensitization in animal models (26), this may be due to the SCFAs produced by these commensal bacteria. In mice, experimental data has shown that increased SCFAs, especially acetate and butyrate, may prevent development of food allergy by way of promoting the tolerogenic effect of CD103^+^ dendritic cells (143). Initial studies have shown that in term infants, total gut SCFA levels are elevated in formula-fed vs. breastfed infants, however acetate levels in particular are highest with exclusive breastfeeding (141, 144). There are no published studies of SCFA levels in human milk.

## Human milk cells

A variety of other factors have yet to be better investigated in terms of the impact on the development of inflammation and immunity. Extremely interesting is recent data suggesting that up to 6% of cells in human milk are stem cells, and mesenchymal stem cells isolated from human milk are potentially reprogrammable to many types of tissue (145, 146). These cells may play a role in development of immune cells, including regulatory T cell, which may suppress antimaternal immunity and lead to microchimerism that induce intestinal tissue repair and immune protection (146). Colostrum is specifically also rich in leukocytes, with breastfed infants being exposed to as much as 10^10^ maternal leukocytes per day, and the role of this exposure in immune development in infants is not yet clear (44). One study of 61 mothers and infants did show that macrophage proportion was significantly smaller in the milk of mothers who had infants with cow's milk allergy compared to mothers who had healthy infants, whereas neutrophil, eosinophil or lymphocyte abundant milk noted significantly more often being received by infants with cow's milk allergy (147). There is still much to learn about the effect of these factors in prevention of allergic disease.

## Dietary antigens

Maternal dietary antigens, including ovalbumin, β-lactoglobulin, gliadin and peanut, have been detected in human milk generally in quantities varying from undetectable levels to 430 ng/ml (148–155). Although their role in inducing symptoms in already sensitized infants has been shown (150), and the ingestion of egg has been associated with immune markers in infants (155), their role in initial sensitization or tolerance development in humans is still debated.

## Conclusions

The immunomodulatory composition of human milk is surprisingly poorly characterized and varies between mothers. The coarse definition of breastfeeding used in epidemiologic studies does not take into consideration the variability in the numerous immunologically active factors in human milk, which may lead to conflicting data regarding the impact of breastfeeding on immune development and downstream implications on development of prevention of allergic disease. Whereas one mother's milk may be rich is immunoprotective factors, another mother's milk may not; however epidemiologic studies do not differentiate between these two very different infant dietary (and microbial) exposures. In addition, randomized controlled trials, with assignments to either breastfeed or not, are lacking, and definitions of breastfeeding and allergic outcomes vary. Unfortunately, most studies have been underpowered for food allergies or not assessed at all due to methodologic problems of making the firm diagnosis.

The studies above suggest that, upon a closer look, the milieu of biomarkers in human milk varies between mothers and the composition may play a function in progression to or prevention against atopy. The impact of human milk biologically active components can be direct or perhaps due to modulation of intestinal microbial composition and function. Most importantly, the factors do not act in isolation, and the study into the impact of a combination or networks of immune factors in human milk on infant microbiome and immune development is still “in its infancy.” Better elucidation of the role of these factors could lead to early targets for treatment and prevention of allergic disease. Further and larger well-characterized studies using prospective cohort data would be extremely helpful in determining the most important factors that likely play a role in development of atopic diseases. The above studies shed a guiding light for future areas of research.

## Author contributions

PR wrote sections including introduction, cytokines, soluble as CD14/TLR, fatty acids, human milk cells, and conclusions. AS and KJ mentors and editors, wrote abstract and sections on HMOs, IgA, and microbiome.

### Conflict of interest statement

The authors declare that the research was conducted in the absence of any commercial or financial relationships that could be construed as a potential conflict of interest.
